# Supplementary material for: Naturally Occurring Osteoarthritis Features and Treatments: Systematic Review on the Aged Guinea Pig Model
Source: Int J Mol Sci. 2022 Jun 30;23(13):7309. doi: 10.3390/ijms23137309 (PMC9266929; doi:10.3390/ijms23137309)
Supplement: Supplementary file 1 [file ijms-23-07309-s001.zip › ijms-1776752-supplementary.pdf]

Excluded studies

As reported in Figure 1, several studies (n = 70) were excluded from the list of accepted included studies because they:

1) were performed in animals other than guinea pigs, such as rats (n = 7), rabbits (n = 6), mice (n = 5), bovines (n = 2), humans (n = 2), mini-pigs (n = 1), sheep (n = 1), and pigs (n = 1);

2) did not treat the pathology of OA, but skeletal muscle injury (n = 3), costal cartilage injury (n = 2), mitochondrial genome sequencing (n = 1), plasma and joint tissue pharmacokinetics (n = 1), arterial hypertension and hyperlipidemia (n = 1), intervertebral disc degeneration (n = 1), hematology (n = 1), hemorrhagic fever (n = 1), muscle afferent neurones disease (n = 1), osteochondral defects (n = 2), ex vivo studies in humans and bovine cartilage (n = 3), finite element (n = 3);

3) treated non-spontaneous OA, but induced through systemic iron overload (n = 1), meniscectomy (n = 7), cranial cruciate ligament excision (n = 3), and anterior cruciate ligament transaction (ACLT) (n = 4);

4) were in vitro studies on chondrocytes (n = 2), synoviocytes (n = 1), immune cells (n = 1), and meniscal cells (n = 2);

5) described equipments for diagnostic purposes, such as a high-resolution small animal ultrasound system with a transducer for image-guided injections (n = 1), and MRI for animal research (n = 2);

6) did not treat knee OA, but temporomandibular one (n = 1).
